# COVID-19 infection rate after urgent versus elective total hip replacement among unvaccinated individuals: A multicenter prospective cohort amid the COVID-19 pandemic

**DOI:** 10.1016/j.amsu.2022.104307

**Published:** 2022-08-01

**Authors:** Seyed Peyman Mirghaderi, Erfan Sheikhbahaei, Maryam Salimi, Seyed Reza Mirghaderi, Negar Ahmadi, Alireza Moharrami, Mehdi Motififard, Seyed Mohammad Javad Mortazavi

**Affiliations:** a Joint Reconstruction Research Center, Tehran University of Medical Sciences, Tehran, Iran; b Students' Scientific Research Center (SSRC), Tehran University of Medical Sciences, Tehran, Iran; c Department of Orthopedic Surgery, Kashani University Hospital, School of Medicine, Isfahan University of Medical Sciences, Isfahan, Iran

**Keywords:** Arthroplasty, COVID-19, Hip injuries, Safety, SARS-CoV-2

## Abstract

**Background:**

Due to the COVID-19 pandemic, hospitals have become unsafe for patients as potential sources of virus transmission. This study aims to determine the COVID-19 infection rate after primary total hip arthroplasty (THA) among unvaccinated patients. THA patients undergoing elective or traumatic (urgent) THA were compared regarding COVID-19 contraction.

**Methods:**

Primary THA patients were prospectively followed from three hospitals in *two great cities* of the country between April 2020 to August 2021. If the patient had suspected COVID-19 symptoms, had a SARS-CoV-2 PCR test from nasopharyngeal and oropharyngeal swabs and/or chest CT scan.

**Results:**

Finally, information was received from 436 patients, including 345 (79.1%) elective and 91 (20.9%) traumatic THAs. Eight patients (1.8%) contracted COVID-19 within a month after THA discharge, and two died due to COVID-19. There was no statistical difference between COVID-19 disease and type of surgery (elective 1.4% versus traumatic 3.3%, P = 0.24). Women (Odds ratio (95% CI) = 8.5 (2.1–35.2), P = 0.01) and those who have heart disease (Odds ratio with Haldane-Anscombe correction ≈ 14.0, P = 0.01) were more likely to contract COVID-19 postoperatively.

**Conclusion:**

In both elective and urgent cases of THA, researchers found that there is not a high risk of contracting the virus during the peri-surgery period. Urgent THA surgeries are comparable to elective THA-with those strict pre-elective surgery protocols-in terms of COVID-19 risk of infection from the hospital stay if appropriate health protocols are followed.

## Highlights

• The COVID-19 pandemic has made hospitals unsafe for elective surgeries.
• Eight patients (from 491, 1.8%) were infected with COVID-19 within a month of THA.
• Four were hospitalized due to severe symptoms.
• Three of 91 traumatic (3.3%) and five among 345 elective cases (1.4%) were infected.
• COVID-19 incidence is two-fold higher in urgent THA patients, but insignificant.

## 1 Introduction

SARS-CoV-2 (COVID-19) became an emerging pandemic in March 2020 and had been rapidly evolving epidemiologically [[1], [2], [3], [4], [5], [6], [7], [8], [9]]. Due to the COVID-19 pandemic, hospitals have become unsafe for patients as potential sources of virus transmission [[10], [11], [12], [13], [14], [15]]. The restriction of total hip arthroplasty (THA) surgeries is one of the consequences of the pandemic. However, increasing patient disability and financial losses to the orthopedic department led to the resumption of elective surgery, even lacking evidence for the safety of these procedures for patients amid COVID-19 pandemics [10,16]. COVID-19 fears have caused some traumatic hip patients to delay seeking medical attention, possibly contributing to their delayed hospital arrival [17].

This study aims to unveil the rate of COVID-19 infection within a month after discharge among unvaccinated patients with primary THA and compare this rate between elective cases performed under strict health protocols and urgent (traumatic) cases. We hypothesized that urgent traumatic cases are more risk due to less severe prevention actions; however, elective cases are safer.

## 2 Materials and methods

### 2.1 Participants and data acquisition

Strengthening The Reporting Of Cohort Studies in Surgery STROCSS criteria were followed in reporting this work [18]. We prospectively collected the data of this study from three hospitals in two cities of *city 1* and *city 2*, with great numbers of patients for THA surgery in *our country*. The subjects were patients who underwent primary THA from April 1st, 2020, to August 1st, 2021, in ** (university-affiliated) and ** (private) hospitals in *city 1* and ** (private) hospital in *city 2*. In all stages of the study, we followed the Institutional and National Research Committee (Approval ID: IR.TUMS.IKHC.REC.1400.509). Written informed consent from all of the participants was obtained before joining to study. During the COVID-19 pandemic, all admitted patients provided written consent regarding the risk of contracting the virus.

Patients' medical records containing demographics, clinical and comorbidity information, and information related to COVID-19 were retrieved and reviewed. Since there was little COVID-19 vaccination coverage during the study period, vaccinated patients were excluded. Patients were contacted weekly for one month and asked about COVID-19 manifestations, and a comprehensive COVID-19-related history was taken from them. Symptoms of COVID-19 such as fever, myalgia, weakness, and gastrointestinal manifestations were asked from them. If the patient had suspected COVID-19 symptoms, had a SARS-CoV-2 PCR test from nasopharyngeal and oropharyngeal swabs and/or chest CT scan. An infectious disease specialist visited COVID-19 suspicious patients to confirm the diagnosis and start treatment. Various parameters concerning their disease were recorded, including the interval between discharge and onset of COVID-19 symptoms, hospital admission, length of stay (LOS), ICU admission, mechanical ventilation, and mortality associated with COVID-19.

### 2.2 Preoperative COVID-19 protocols

There are two different steps in hospitals to screen for preoperative COVID-19 infection. First, they received a complete medical history (including recent symptoms of COVID-19 and a history of contact with people with COVID-19 infection). Then they measured the body temperature with a digital thermometer and oxygen saturation by pulse oximetry. All three hospitals conducted these routine assessments before TJR surgery on all patients and continued checking patients every day during hospitalization. A second part of the testing was laboratory and imaging examinations, which varied between hospitals. In the city1, COVID-19 was tested twice with RT-PCR: 2–3 days before the operation and on the day of surgery. Patients in city2 did not undergo COVID-19 testing by RT-PCR, and chest X-rays (CXR) instead were used for screening. The preoperative COVID-19 screening protocol was similar for elective and traumatic cases. The COVID-19 clinic then investigated those suspected of the virus (such as those with COVID-19-related symptoms or airspace opacities in CXR). Once the infectious disease service ruled out COVID-19, the patient was considered for surgery.

Patients with trauma first arrived at the hospital to emergency services. After stabilizing vital signs and treating emergency injuries, they prepared to perform THA in the early stages. Patients referred for trauma spent more time in the emergency room, which is a possible site of infection with COVID-19. However, elective THA patients were hospitalized just a night before the surgery in the orthopedic ward. As a precaution, all patients were asked to wear protective masks and handwash, not have any visitors except for one companion, and were put in rooms with a maximum capacity of three people.

### 2.3 Statistical analysis

All statistical calculations were performed in IBM SPSS (version 22) software. Comparing the qualitative variables across the groups (positive versus negative COVID-19) was done using Fisher's exact and chi-squared tests. Based on the normality of the data, quantitative variables were compared using the independent sample T-test. P-value <0.05 was considered significant (2-sided).

## 3 Results

A total of 533 primary THA consecutive patients participated in the study, of Which 40 individuals were excluded due to COVID-19 vaccination; 436 patients (out of 493 unvaccinated patients, 88.4%) were responsive by the end of the study. The mean age of all patients was 51.4 ± 17.4, and 55.7% (243) were female. A total of 91 patients (20.9%) had urgent THA surgery, all for traumatic fractures. Forty patients (9.2%) had a history of COVID-19 infection before surgery, with a mean time interval of 3.2 months (range 1–17 months). Both elective and traumatic THA patients were comparable before the surgery in terms of gender, body mass index (BMI), city, hospital type, prior COVID-19 infection, kidney disease, and history of cancer (Table 1) (P > 0.05). However, the traumatic patients were older (61.1 ± 17.0 vs. 48.9 ± 16.4, P < 0.001), longer LOS (6.1 ± 5.3 vs. 4.5 ± 3.6, P = 0.02), and more likely to have comorbidities (diabetes mellitus, hypertension, pulmonary disease, cardiac disease) (P < 0.05). The immune disease was more frequent among the elective group (13.0% vs. 4.4%, P = 0.02).

**Table 1:** Comparing the demographic and clinical information of two included groups of patients (elective vs. traumatic THA).

	Total	Type of THA		P*
	(N = 436)	Elective (N = 345, 79.1%)	Traumatic (N = 91, 20.9%)	
Gender				
**Female**	243 (55.7%)	199 (57.7%)	43 (47.3%)	0.07
**Male**	193 (44.3%)	146 (42.3%)	48 (52.7%)	
Age/year **(mean ± SD)**	51.4 ± 17.4	48.9 ± 16.4	61.1 ± 17.0	**>0.001**
BMI	25.8 ± 4.8	26.1 ± 4.9	25.1 ± 4.2	0.08
City				
*City 2*	134 (30.7%)	110 (31.9%)	24 (26.4%)	0.31
*City 1*	302 (69.3%)	235 (68.1%)	67 (73.6%)	
Hospital length of stay for surgery/days **(mean ± SD)**	4.7 ± 3.9	4.5 ± 3.6	6.1 ± 5.3	**0.02**
Hospital type				
**Private**	200 (45.9%)	166 (48.1%)	34 (37.4%)	**0.07**
**Public**	236 (54.1%)	179 (51.9%)	57 (62.6%)	
Prior COVID-19 infection				
**Yes**	40 (9.2%)	27 (7.8%)	13 (14.3%)	0.07
**No**	396 (90.8%)	318 (92.2%)	78 (85.7%)	
Comorbidity				
**Diabetes mellitus**	52 (11.9%)	31 (9.0%)	21 (23.1%)	**>0.001**
**Hypertension**	98 (22.5%)	66 (19.1%)	32 (35.2%)	**0.001**
**Pulmonary disease**	22 (5.0%)	13 (3.8%)	9 (9.9%)	**0.02**
**Kidney disease**	17 (3.9%)	14 (4.1%)	3 (3.3%)	0.74
**Cardiac disease**	49 (11.2%)	33 (9.6%)	16 (17.6%)	**0.03**
**Immune disease**	49 (11.2%)	45 (13.0%)	4 (4.4%)	**0.02**
**Cancer**	14 (3.2%)	10 (2.9%)	4 (4.4%)	0.47

*P-value: Chi-square test or independent *t*-test.

**Bold** values: significant p-value.

THA, total hip arthroplasty; OA, osteoarthritis; DDH, developmental dysplasia of the hip; AVN, avascular necrosis; BMI, body mass index.

Eight patients (1.8%) became infected with COVID-19 within a month after surgery; four were hospitalized due to severe symptoms. Three patients died after surgery, two due to the COVID-19 and one non-related to the COVID-19 infection. Table 2 summarizes the information of all included patients with complete follow-up categorized by COVID-19 status, and Table 3 shows infected cases with COVID-19 within a month after discharge from THA hospitalization. Female gender was a risk factor for disease (P = 0.01, Odds ratio (95% CI) = 8.5 (2.1–35.2)). Table 2 shows the comorbidities of patients. They are not statistically significant between COVID-19 positive and negative groups, except for heart disease (P = 0.01, Odds ratio with Haldane-Anscombe correction ≈ 14.0).

**Table 2:** Demographic and clinical information of included patients (mean ± SD or n, %).

	Total	COVID-19 within one-month post-surgery		P*
	N = 436, 100%	Yes (N = 8, 1.8%)	No (N = 428)	
Urgent/elective				
**Elective**	345 (79.1%)	5 (62.5%)	340 (79.4%)	0.37
**Urgent (traumatic)**	91 (20.9%)	3 (37.5%)	88 (20.6%)	
Gender				
**Female**	243 (55.7%)	8 (100%)	235 (54.9%)	0.01* OR (95% CI) ≈ 14.0
**Male**	193 (44.3%)	0	193 (45.1%)	
Age**/year**	51.4 ± 17.4	59.9 ± 29.0	51.3 ± 17.0	0.43
BMI	25.8 ± 4.8	25.9 ± 4.7	24.9 ± 5.7	0.57
City				
*City 2*	134 (30.7%)	3 (37.5%)	131 (30.6%)	0.71
*City 1*	302 (69.3%)	5 (62.5%)	297 (69.4%)	
Hospital length of stay for surgery**/days**		5.2 ± 2.9	4.7 ± 3.9	0.76
Hospital type				
**Private**	200 (45.9%)	2 (25%)	198 (46.3%)	0.23
**Public**	236 (54.1%)	6 (75%)	230 (53.7%)	
Prior COVID-19 infection				
**Yes**	40 (9.2%)	1 (12.5%)	39 (9.1%)	0.54
**No**	396 (90.8%)	7 (87.5%)	389 (90.9%)	
Indication for surgery				
**Primary OA**	157 (36.0%)	3 (37.5%)	154 (36.0%)	0.97
**AVN**	97 (22.2%)	1 (12.5%)	96 (22.4%)	
**Inflammatory arthritis**	12 (2.6%)	0	12 (2.8%)	
**DDH**	74 (17.0%)	1 (12.5%)	73 (17.1%)	
**Haemophilia and others**	5 (1.1%)	0	5 (1.2%)	
**Traumatic fractures**	91 (20.9%)	3 (37.5%)	88 (20.6%)	
Comorbidity				
**Diabetes mellitus**	52 (11.9%)	1 (12.5%)	51 (11.9%)	1.00
**Hypertension**	98 (22.5%)	3 (37.5%)	95 (22.2%)	0.39
**Pulmonary disease**	22 (5.0%)	1 (12.5%)	21 (4.9%)	0.34
**Kidney disease**	17 (3.9%)	0	17 (4.0%)	1.00
**Cardiac disease**	49 (11.2%)	4 (50.0%)	45 (10.5%)	0.01* - OR (95% CI) = 8.5 (2.1–35.2)
**Immune disease**	49 (11.2%)	0	49 (11.4%)	0.61
**Cancer**	14 (3.2%)	0	14 (3.3%)	1.00

*P-value: Fisher-exact test or independent *t*-test.

**Haldane-Anscombe correction.

OR = Odds ratio.

**Bold** values: significant p-value.

OA, osteoarthritis; DDH, developmental dysplasia of the hip; AVN, avascular necrosis.

**Table 3:** Characteristics of COVID-19 positive cases within a month after THA discharge.

Patient number	Gender	COVID-19 symptoms interval after the surgery (days)	Hospitalization for COVID-19 day	ICU admission (days)	Death	age	City	Indication	Urgent/elective
**1**	F	14	0	0	No	17	City 1	DDH	Elective
**2**	F	20	0	0	No	72	City 1	Trauma	Urgent
**3**	F	25	4	4	Yes	84	City 2	Trauma	Urgent
**4**	F	4	7	0	No	87	City 2	Trauma	Urgent
**5**	F	3	8	5	No	39	City 1	AVN	Elective
**6**	F	5	0	0	No	25	City 1	OA	Elective
**7**	F	8	4	4	Yes	90	City 1	OA	Elective
**8**	F	1	0	0	No	65	City 2	OA	Elective

OA, osteoarthritis; DDH, developmental dysplasia of the hip; AVN, avascular necrosis.

We had 91 traumatic patients, who underwent primary urgent THA, 3 of whom were infected (3.3%(, and five were infected among 345 elective cases (1.4%). Although COVID-19 incidence is two-fold higher in urgent THA patients, this difference is not statistically significant (P = 0.24). There was no statistically significant difference in COVID-19 infection prevalence after one month of discharge in terms of different cities (P = 0.71), indications for surgery (P = 0.97), and mean age of patients (P = 0.43, Table 2).

## 4 Discussion

Health systems have undergone many changes with the global onset of the COVID-19 epidemic [[19], [20], [21], [22]]. Hospitals postponed non-urgent surgeries, including arthroplasty. However, hospitals resumed elective THA under strict health protocol. In this study, we compared the incidence of COVID-19 among unvaccinated patients who underwent primary, urgent THA surgery due to traumatic fractures with patients who had primary elective THA surgery. While trauma patients spend time in the hospital emergency ward, which increases the risk of COVID-19 infection, elective patients were already aware of their surgery and took extra care and preventive actions. We found out that of 436 THA patients, COVID-19 has infected as low as 1.8% (n = 8) within a month after THA discharge. Three (3.3%) of the traumatic THA patients were infected with COVID-19, nearly twice as many as elective THA patients (1.4%), but this difference is not statistically significant (P = 0.24). Female gender and heart disease were risk factors for Infection with COVID-19. There was no significant difference between the type of surgery, age, indication for surgery, hospital type, and city with the risk of COVID-19.

We previously reported the incidence of symptomatic COVID-19 in unvaccinated patients who underwent elective TJA within one month of discharge in our country (April 2020–April 2021) [23]. COVID-19 occurred in 2.4% of patients within one month of TJA discharge, similar to the 1.8% infection rate among elective and urgent THA patients. Scarce studies examined the incidence of COVID-19 separately in the elective and urgent settings of surgeries. The study by Agrawal et al. found that of 167 elective joint replacement surgery cases, one patient (0.6%) was infected with COVID-19 early postoperatively. COVID-19 incidence was significantly higher among the urgent or emergency arthroplasty procedures performed during the study period (6 out of 57, 10.5%) than elective procedures [24]. Our study supports this finding; however, the comparison was not statistically significant.

Most previous studies deemed safe resumption of surgeries under safety protocols [[25], [26], [27], [28], [29], [30], [31]]. In a study in the UK, Infection with COVID-19 after hip and knee arthroplasty was comparable to the general population (0.5%), and LOS is the only risk factor [25]. They revealed that up to 2-fold higher (1%) is the accurate rate if count patients do not present to the healthcare services similar to the general population. The mortality rate was negligible (0.9%) [25]. Nevertheless, Clement et al. examined postoperative mortality among orthopedic and trauma surgery patients. Mortality was significantly higher in the group that tested positive before surgery, and female gender and older ages were risk factors. Sixty of the 62 positive COVID-19 tests were in the emergency surgery group [32].

Stoneham et al. showed a 2% COVID-19 incidence during THA for trauma amid the disease peak in the UK. This study did not compare elective surgery, but it suggested restarting it based on its results [33]. Skibicki et al. revealed the incidence of COVID-19 after nonelective hip and knee surgery at 3.4%, none of them was in arthroplasty patients (0 out of 73) [26]. Thus the low incidence of COVID-19 is suspected during the surge of the COVID-19 pandemic.

A prospective study by Balieiro et al. evaluated 300 patients undergoing bariatric surgery who were all negative for COVID-19 pre-operatively, and there was no report of postoperative COVID-19 infections or deaths [34]. As a result, the authors recommend a preoperative screening protocol that includes a questionnaire regarding the related symptoms and an RT-PCR test for those undergoing elective surgery during the pandemic. Gehrke et al. evaluated the preoperative screening protocol using the Parvizi et al. questionnaire, which is included in the International Consensus Group (ICM) guidelines for preoperative screening [35,36]. Symptoms suggestive of COVID-19, as well as any contact with the infected patients, were assessed by the questionnaire. No patient categorized at low risk by Gehrke et al. tested positive for COVID-19. It thus appears that strict screening protocols do little for patients. The authors, therefore, believe that their study protocol was sufficient to prevent further consequences from COVID-19 preoperative screening.

We have some limitations in this study, including a small sample size - insufficient for statistically significant results, different hospitals with unidentical protocols, considering only symptomatic cases of COVID-19, and about 11% of patients lost to follow-up. Due to extensive lockdowns and cancellations of numerous elective surgeries, our sample of patients who underwent TJA was relatively small, making it challenging to compare the two groups with high power. To address this limitation, we added more centers.

## 5 Conclusion

Despite widespread vaccination, the emergence of new strains of COVID-19 has put us at war with the virus. We need to adjust to this new reality to continue our daily lives. According to study findings, there is no high risk of contracting the virus during the THA peri-surgery period in elective and urgent cases. Urgent THA surgeries are comparable to elective THA-with those strict pre-elective surgery protocols-in terms of COVID-19 risk of infection from the hospital stay if appropriate health protocols are followed. When interpreting the results, care should be taken since there is no way to eliminate the risk of infection with COVID-19 in healthcare settings.

## Ethical approval

The present study methodology was reviewed and approved by the Institutional Review Board (IRB) of the Tehran University of Medical Science, and the study was declared to be of no ethical concern by the ethics committee of the mentioned university (Approval code: IR.MUI.MED.REC.1400.390).

## Sources of funding

None. The study has no sponsors.

## Author contribution

SP.M designed the study, edited the manuscript, and analyzed the data. SR.M helped in the study design and edited the manuscript. E.Sh, N.A, M.S, and A.M helped gather data and write the draft. M.M and SMJ.M introduced the idea, performed surgeries, guided the authors, and revised the final draft.

## Consent

Written Informed consent was obtained from all the patients to publish this study and accompanying data. A copy of the written consent is available for review by the Editor-in-Chief of this journal on request.

## Registration of Research Studies

Not applicable.

## Guarantor

Seyed Mohammad Javad Mortazavi M.D.

## Provenance and peer review

Not commissioned, externally peer-reviewed.

## Declaration of competing interest

The authors have no relevant financial or non-financial interests to disclose.
